# Validation of electronic performance and tracking systems EPTS under field conditions

**DOI:** 10.1371/journal.pone.0199519

**Published:** 2018-07-23

**Authors:** Daniel Linke, Daniel Link, Martin Lames

**Affiliations:** Chair of Performance Analysis and Sports Informatics, Technical University Munich, Munich, Germany; Universita degli Studi di Verona, ITALY

## Abstract

The purpose of this study was to assess the measurement accuracy of the most commonly used tracking technologies in professional team sports (i.e., semi-automatic multiple-camera video technology (VID), radar-based local positioning system (LPS), and global positioning system (GPS)). The position, speed, acceleration and distance measures of each technology were compared against simultaneously recorded measures of a reference system (VICON motion capture system) and quantified by means of the root mean square error RMSE. Fourteen male soccer players (age: 17.4±0.4 years, height: 178.6±4.2 cm, body mass: 70.2±6.2 kg) playing for the U19 Bundesliga team FC Augsburg participated in the study. The test battery comprised a sport-specific course, shuttle runs, and small sided games on an outdoor soccer field. The validity of fundamental spatiotemporal tracking data differed significantly between all tested technologies. In particular, LPS showed higher validity for measuring an athlete’s position (23±7 cm) than both VID (56±16 cm) and GPS (96±49 cm). Considering errors of instantaneous speed measures, GPS (0.28±0.07 m⋅s^-1^) and LPS (0.25±0.06 m⋅s^-1^) achieved significantly lower error values than VID (0.41±0.08 m⋅s^-1^). Equivalent accuracy differences were found for instant acceleration values (GPS: 0.67±0.21 m⋅s^-2^, LPS: 0.68±0.14 m⋅s^-2^, VID: 0.91±0.19 m⋅s^-2^). During small-sided games, lowest deviations from reference measures have been found in the total distance category, with errors ranging from 2.2% (GPS) to 2.7% (VID) and 4.0% (LPS). All technologies had in common that the magnitude of the error increased as the speed of the tracking object increased. Especially in performance indicators that might have a high impact on practical decisions, such as distance covered with high speed, we found >40% deviations from the reference system for each of the technologies. Overall, our results revealed significant between-system differences in the validity of tracking data, implying that any comparison of results using different tracking technologies should be done with caution.

## Introduction

Electronic performance and tracking systems (EPTS) primarily track player (and ball) positions and have become one of the most important components to monitor a player’s overall external (locomotor) load [1]. In particular, semi-automatic multiple-camera video systems (VID), radio-based local positioning systems (LPS) and global positioning systems (GPS) have become indispensable core technologies for assessing the physical and tactical behaviour of both training and competition [2, 3]. As a matter of fact, it is not uncommon for some players to be tracked by two or three different EPTS during a regular week, considering that GPS systems and/or LPS systems are often used during training sessions, while most teams obtain positional data from official matches from semi-automatic camera systems [4]. Consequently, validity, interchangeability and agreement between different EPTS are of key importance to allow for a substantiated assessment of a player’s overall locomotor load and to integrate the data of different systems in a meaningful way.

A review of the literature on the subject of EPTS’ validity reveals that previous studies differ with regard to the number of tested core technologies (single technology [5–15] vs. multiple technology studies [3, 16, 17]), the choice of exercises (predefined movement patterns [3, 5, 7, 11, 13, 14, 17, 18] vs. complex and free movements scenarios [6, 8, 19]), and, most importantly, the utilized criterion method. The most commonly used criterion methods include predefined movement circuits with known spatial arrangements (to evaluate distance measurement accuracy) [3, 7, 11, 12, 18], timing gates (to evaluate average speed) [3, 5, 7, 9, 12, 18], and radar/laser-based speed measurements for the evaluation of instantaneous running speed [10, 13, 17].

However, all these methods have specific drawbacks. First, distance references that are based on predefined running circuits are inevitably susceptible to errors introduced by the participants (e.g. errors introduced by postural sway or the difficulty for participants to follow the marked course as precisely as possible [7]). Second, timing gates are only of limited suitability as a speed reference [20], the reason for this being that this approach only determines average speed based on limited sampling points [21]. Third, while radar/laser guns are capable of measuring the instantaneous speed of an object with high accuracy, they are suitable only when it comes to validating linear running movements without changes in direction [17].

Therefore, the actual positional data obtained by EPTS should ideally be compared with the instantaneous positional and speed data of a two or three-dimensional reference system with known error estimates [8]. However, to our knowledge, merely four validation studies used a kinematic analysis approach to evaluate the validity of EPTS. Specifically, Duffield et al. [6] and Vichery et al. [19] used a VICON motion analysis system to validate GPS systems in field-based team sports, while Ogris et al. [8] and Stevens et al. [22] investigated the accuracy of a radar-based LPS-system during soccer-specific movements. Limitations of the aforementioned studies include a lack of instantaneous accuracy measures for both speed and position (rather than merely average differences of the mean aggregated data) [6, 19, 22], missing information on the specific data processing steps [6, 19], a lack of realistic game scenarios [6, 19, 22], an insufficient size of the test area [6, 19, 22], as well as a lack of direct comparison between different technologies [6, 8, 19, 22].

A review of the literature further reveals that previous EPTS validation studies can be divided into three categories, according to the examined parameters. The first category contains studies that analyzed position accuracy (spatial coordinates) [8, 17]. Others examined the accuracy of instantaneous speed and acceleration data [10, 23]. Errors in this category could result from either a poor quality of position data or inadequate processing algorithms [21]. Eventually, an accumulation of errors in the first two categories can lead to errors in the third category: key performance indicators (KPI) that are aggregated from the continuous data (e.g. distance covered, mean or peak speed, peak accelerations, etc.) [6, 19, 22]. Consequently, aiming at a comprehensive accuracy assessment of EPTS requires comparisons in three different categories, because in each category different problems could occur and different accuracy demands are to be met.

Furthermore, up-to-date information on the spatial accuracy of sport-specific GPS technologies is still missing in the current literature. Considering the fact that various studies made use of GPS-based spatial coordinates to answer relevant scientific questions [24, 25], as well as the fact that several commercial GPS-systems determine distance via positional differentiation and speed via Doppler shift [21], information on the spatiotemporal accuracy of sport-specific GPS-systems is still scarce.

Therefore, the purpose of the current study was to assess the accuracy of the most commonly used tracking technologies in professional team sports under field conditions (i.e., semi-automatic multiple-camera video technology, radar-based LPS technology, and GPS technology). Measures of each technology were compared to that of a reference system (*VICON*). This was done for test runs along predefined tracks, shuttle runs, and small sided games. The results could contribute to an improved understanding of performance parameters provided by EPTS.

## Methods

### Participants

Fourteen male soccer players (age: 17.4±0.4 years, height: 178.6±4.2 cm, body mass: 70.2±6.2 kg) playing for the German Bundesliga team FC Augsburg participated in the study. Prior to participation, all players received comprehensive verbal and written explanations of the study, which was conducted within a period of two consecutive days. On each single day, 10 players participated. On the second day, four players from the first day had to be substituted. Therefore, fourteen different individual players participated in total. Voluntarily signed informed consent to wear GPS/LPS sensors and VICON markers and to participate in the collection of spatiotemporal tracking data was provided by both the players and their parents. Institutional board approval for the study was obtained from the Ethics Commission of the Technical University of Munich. To ensure confidentiality, all performance data were anonymized. This study conformed to the recommendations of the Declaration of Helsinki.

### Validated systems

The following EPTS were included in the validation study:

**Video Technology (VID)**. STATS SportVU (three-camera HD system, cameras: 3 x BASLER acA2500-14gc, 2560*1500 pixels, 16 frames per second). Software: STATS SportVU version 2.12.0, build # 12351. The camera elevation angle ranged from 22° (close sideline) to 11° (rear sideline).

**Global Positioning System (GPS)**. GPSports (GPSports Sports Performance Indicator (SPI) Pro X, Canberra, Australia). This version of the SPI Pro provides raw position, instant speed and distance data at 15 Hz (5 Hz interpolated to 15 Hz). Software: Team AMS firmware: R1 2015.10. All GPS devices were activated 15 min prior to the data collection to allow the acquisition of satellite signals. Unfortunately, horizontal dilution of precision (HDoP) information cannot be retrieved with the provided Team AMS software. After making a request to the manufacturer in this regard, we were informed that the internal code automatically rejects data with HDoP values >4, which is well below the maximum value of 50 [26].

**Local Positioning System (LPS)**. Inmotio (LPM system, 1 kHz, Inmotio Object Tracking BV, Amsterdam, Netherlands). Software: Inmotio Client, firmware: v3.7.1.153. 11 base stations were set up and calibrated under the supervision of an expert of the Inmotio company. During data collection, 22 transponders were activated to simulate a real match situation in terms of the number of transponders that were active at the same time, which resulted in an individual sampling rate of 45.45 Hz (1 kHz/22 transponders). LPM data was filtered with the integrated weighted Gaussian average filter set at 85%, as recommended by the manufacturer.

To ensure optimal device positioning on the body and minimization of crosstalk between GPS and LPS, athletes wore only one device of each system simultaneously. Using the harness provided by the manufacturers, GPS devices were positioned on the upper thoracic spine between the scapulae. LPS devices were worn in a vest containing a transponder located on the back that was connected to two antennas, one on top of each shoulder. The position of the athlete is then calculated as the spatial center of both antennas (manufacturer information).

### Reference system

#### VICON system specifications

An infrared camera-based motion capture system (VICON, Oxford, UK) was utilized to determine criterion position, speed, and acceleration data. The setup comprised 33 cameras in total (six *Vantage 5* cameras (16.0 mm), nine *Bonita* cameras (8.5 mm), 12 *MX T10-S* (8.5—12.5 mm) and six *MX T10* (8.5—12.5 mm), Software: Nexus, Version 2.3). Retro-reflective markers with a diameter of 38.0 mm were used to assure stable recognition of the markers within the entire measurement area (30.0 x 30.0 m, 900.0 m^2^).

#### VICON measurement accuracy

To demonstrate the spatial accuracy of the applied VICON setup, a rigid calibration object was moved through the VICON area, spiraling from the center to the edges of the measurement volume. As the markers on the calibration object remained at accurately known distances to each other at any given time, the distances between the markers that are delivered by the VICON software, which were calculated in retrospect, can be used to describe the crucial aspect of measurement accuracy (see S1 Dataset). Overall, the average error of the calibrated VICON setup was 0.0 mm (SD = 1.0 mm, 95% CI [-1.9 mm, +2.0 mm]), resulting in an RMSE of 1.0 mm at a frequency of 100 Hz.

#### Comparison criteria: Center of mass (COM)

Under the assumption that each EPTS endeavors to detect the position of the human body as a whole, the center of mass (COM) (or rather the XY-position of the body’s center that is projected on the ground plane) was considered a valid criterion measure. However, in the case of wearable tracking devices, the systems actually detect the position of the sensors that are fixed to the players (usually attached between the shoulder blades or on top of the shoulders). In video-based systems, objects are tracked by image segmentation using different techniques of image recognition [27]. Typically a rectangle is identified enclosing segmented parts of the player, and a weighed estimate of the body parts locates the body’s center.

**Fig 1:**
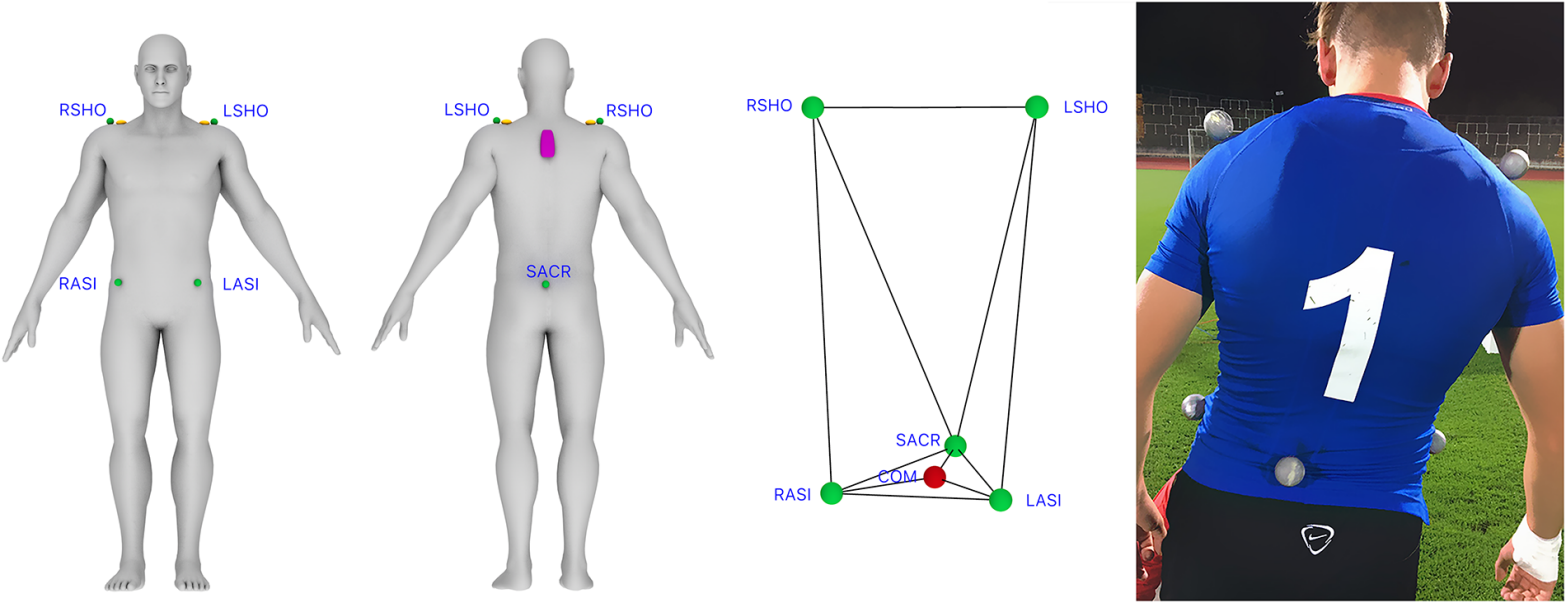
Device positions. Device positions on the human body. VICON markers (green), GPS receiver between the scapulae (purple), LPS antennas on top of both shoulders (orange), center of mass COM (red).

Eventually, the choice of the most suitable reference position on the human body should not be prescribed by the technological prerequisites of the respective EPTS, but rather by biomechanical considerations. We, therefore, advocate the idea that the ultimate reference position for each EPTS should be the COM, irrespective of where the respective transponder/receiver is attached to the human body. To estimate COM, five adhesive marker mounts were glued on each participant’s skin (right shoulder (RSHO), left shoulder (LSHO), left anterior superior iliac spine (LASI), right anterior superior iliac spine (RASI), and sacrum (SACR)) (see Fig 1). The reflective markers were then fixed to the mounts through a tight-fitting compression shirt. COM is then estimated by means of the reconstructed pelvis method [28], defined as the spatial center of the RASI, LASI, and SACR.

### Venue and satellite reception

**Fig 2:**
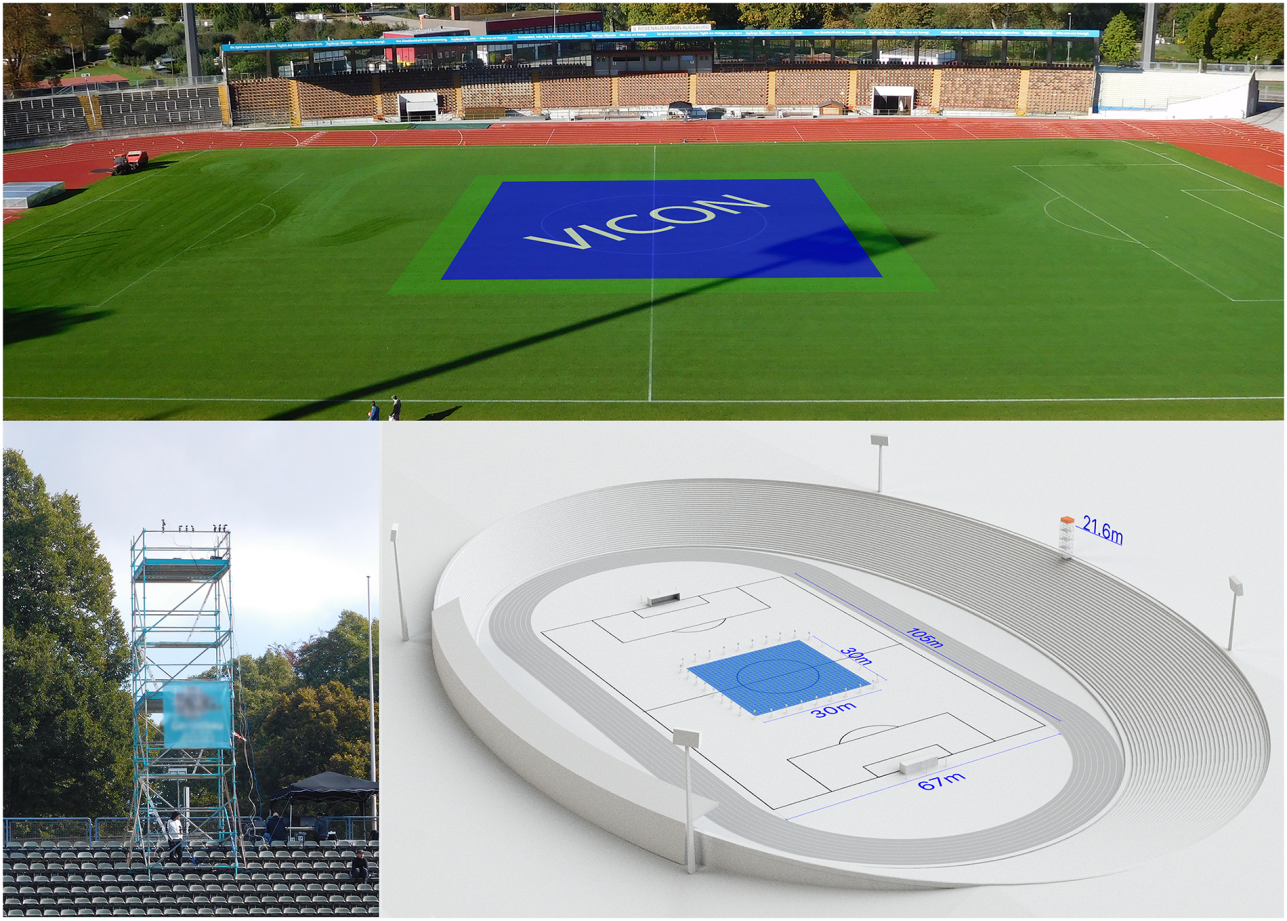
Venue. (Top) VICON test location on the pitch; (bottom right) scaled 3D model of the Rosenaustadion Augsburg. VICON area (blue), VID camera position (orange) at 21.6 m height and 82.0 m distance from the center spot. Pitch size: 105.0 x 67.0 m; (bottom left) additional camera platform.

Measurements took place at Rosenaustadion (Augsburg, Germany). This particular stadium is characterized by low stands (12.0 m maximum height at 50.0 m distance from the sideline). In addition, the pitch (105.0 m x 67.0 m) is surrounded by a tartan track. To meet the standard requirements for the camera system (sufficient height to obtain the required viewing angle), an additional platform had to be built on top of the stands (see Fig 2). During the entire measurement period, the number of connected GPS satellites was 10.1 ± 0.8, which is in the range of previous validation studies (e.g. 8 ± 1 [19], 9.5 ± 2 [3] and 12.3 ± 0.3 [13]). Thus, for all technologies involved, the minimum requirements were met. Data was recorded after sunset using floodlights. The weather was dry and windless with temperatures around 8°Celsius.

### Exercises

#### Sport-specific course (SSC)

**Fig 3:**
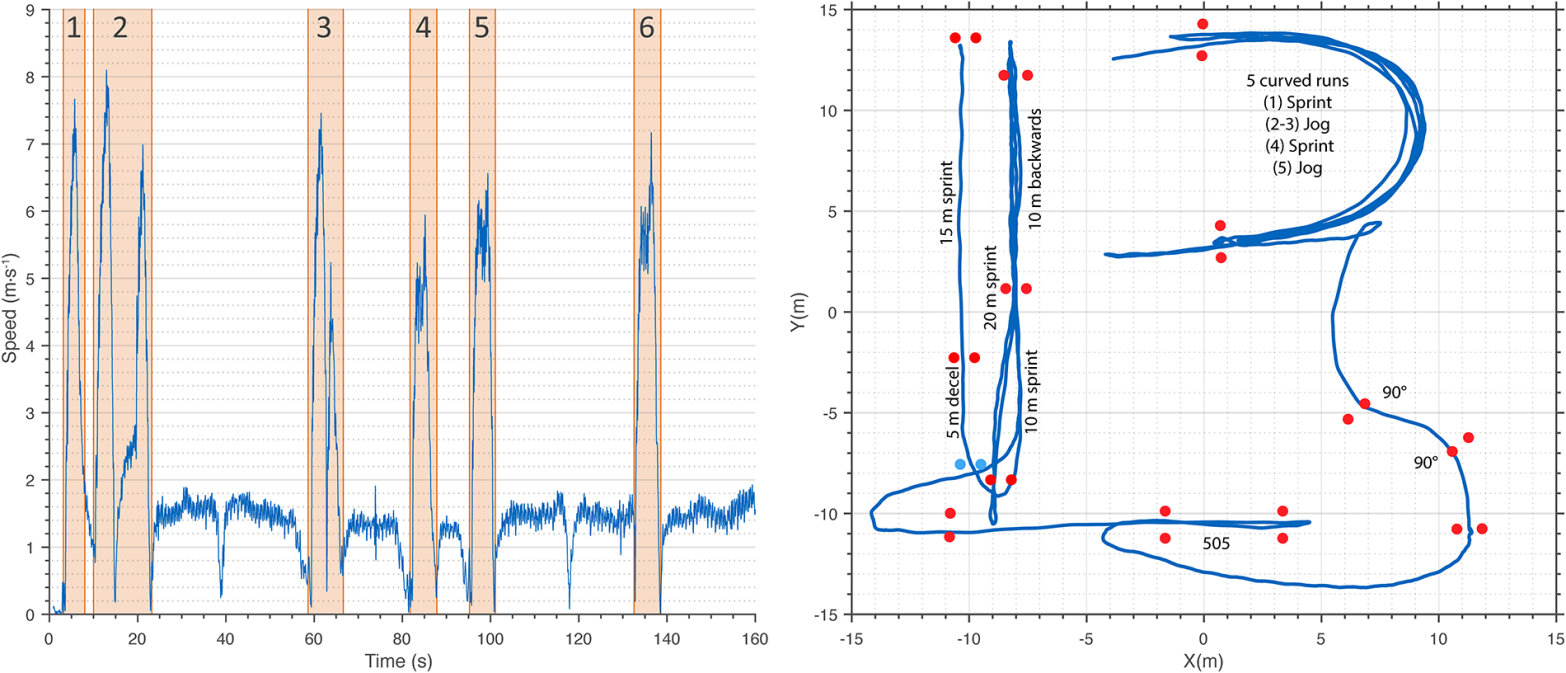
Sport-specific course (SSC) illustrated with exemplary VICON data. **Left**: chronological sequence of movement patterns (1 = 15 m sprint into 5 m deceleration; 2 = 20 m sprint into 10 m backward running into 10 m forward running; 3 = 505 agility test; 4 = two rapid 90° turns; 5&6 = curved runs toward and away from the camera). **Right**: spatial representation of movement patterns. Starting position in the top-left corner.

A predefined circuit with prescribed movement intensities (Fig 3) was used to analyze elementary movements under controlled conditions, e.g. curved runs and runs with sharp turns. Within each trial, six distinct elementary movement patterns were performed: (1) 15 m sprint into 5 m deceleration, (2) 20 m sprint into 10 m backward running into 10 m forward running, (3) 505 agility test, (4) two rapid 90° turns, (5&6) curved runs toward and away from the camera (see S2 Video). The beginning and end of each individual section was marked with two flat pylons, which in turn were equipped with reflective VICON markers. This enabled us in hindsight to detect the starting and endpoint of each section by means of the players’ XY-position (a player was located within/outside a certain section if his COM crossed the line between the two start/end points).

#### 20 m shuttle run test (SHU)

Players repeatedly ran 20 m shuttles with 180° changes of direction at 11 km⋅h^-1^ for a period of two minutes. Subjects ran in groups consisting of ten players each. The shuttle run test was performed to obtain controlled test conditions including change of directions.

#### Small-sided game (SSG)

Finally, exercises with the highest ecological validity are matches that take place on a full-sized pitch. Unfortunately, to the best of our knowledge, a gold standard for full pitch testing does not exist to date. Therefore, the best possible alternative are SSGs with fewer players competing on a smaller sized field. In our case, 5vs5 small-sided games were played, without goals, as collective possession play (see S2 Video). The format of the game-play comprised repeated 2-min bouts interspersed with 1-min of passive rest. Each drill was performed in a continuous regime, under the supervision, coaching, and motivation of the coaches to maintain a high work-rate. The ball was always available owing to prompt replacement any time it was hit out of the measurement area.

### Data analysis

#### Parameters for analysis

As indicated in the introduction, the validation of EPTS should be implemented through analysis of (i) position data, (ii) instant speed and acceleration data, and (iii) KPIs. It should also be noted here, that modern GPS systems derive speed and acceleration data based on the Doppler shift effect, instead of differentiation of position data [21]. We procured fundamental and derived data from the export option of each tracking system (XY-data, instant speed, and acceleration). Instead of using the KPIs as provided by the manufacturers’ proprietary software, we deliberately decided to re-calculated these metrics, allowing us to use exactly the same algorithms for all tested systems. Manufacturer proprietary software often use data-processing algorithms that are subject to intellectual property protection, and their specific algorithms are not disclosed to the end user [21]. Therefore, to achieve a transparent validation procedure, and to facilitate appropriate interpretation and replication by others, it was decided to independently calculate the KPIs based on the provided raw data. Running intensities were divided into the following speed thresholds: standing (<1 km⋅h^-1^), low speed (≥1 to <6 km⋅h^-1^), moderate speed (≥6 to <15 km⋅h^-1^), elevated speed (≥15 to <20 km⋅h^-1^), high speed (≥20 to <25 km⋅h^-1^), and very high speed (≥ 25 km⋅h^-1^). Peak speed was defined as the highest measured speed value. High acceleration and deceleration thresholds were set at ≥3 m⋅s^-2^, and ≤3 m⋅s^-2^, respectively.

#### Data processing

To produce an evenly sampled time series among the systems prior to accuracy analysis, each data set was up-sampled to 100 Hz. The timing offset between the data sets was estimated by means of a cross-correlation procedure. Each coordinate system was then aligned with the VICON coordinate system via a generalized Procrustes analysis (GPA, euclidean similarity transformation, i.e. translation and rotation). After spatial and temporal synchronization of all systems involved, the VICON time code served as the ultimate reference for detecting the EPTS’s start and end points of the respective exercise/section.

Data processing of raw VICON data consisted of filtering using a 4th order 10 Hz Butterworth low pass filter. Gaps in the data of 1 to <10 ms were filled using spline interpolation. Gaps that were ≥10 ms were excluded from analysis. XY-positions for spatial accuracy analysis were directly derived from the 100 Hz VICON data. The third dimension (Z-coordinate) was neglected in the calculations.

**Fig 4:**
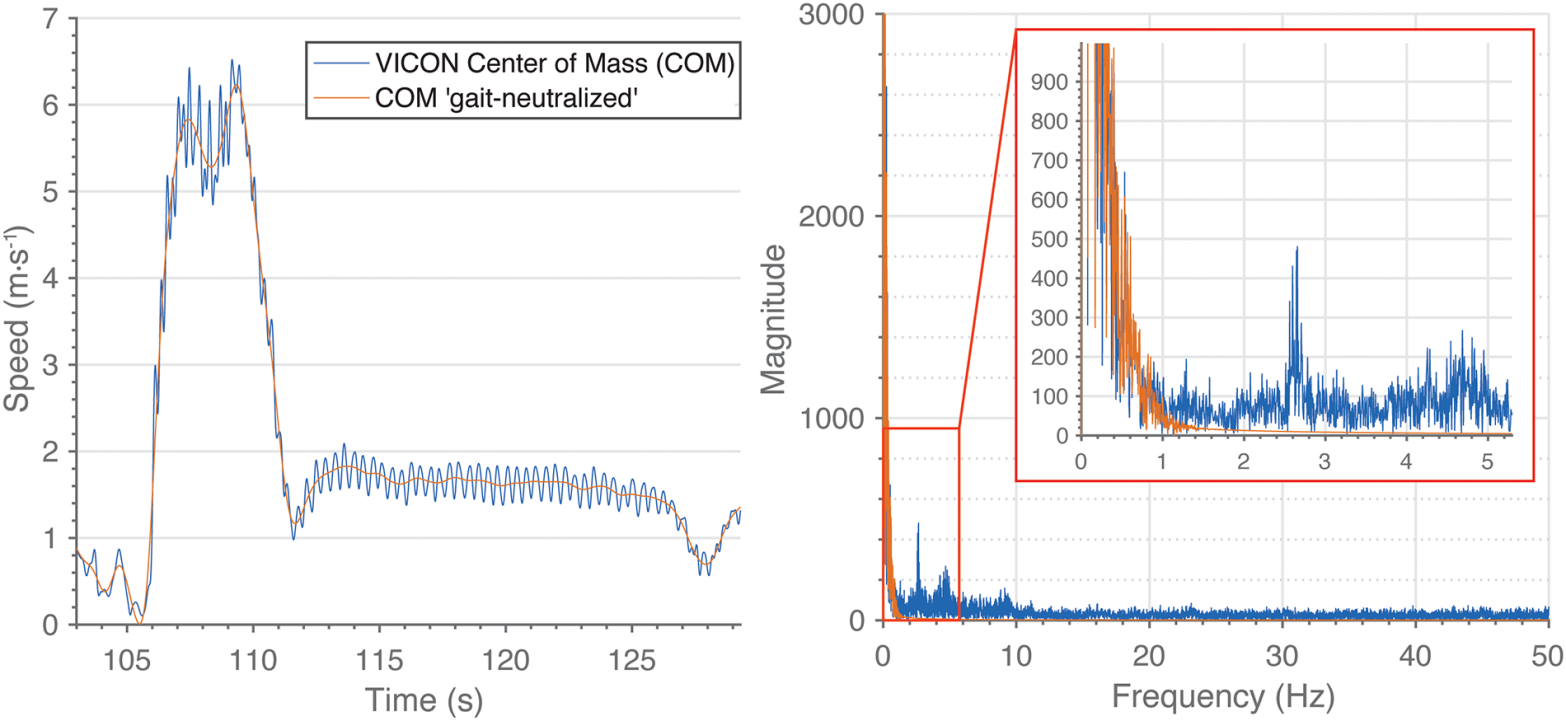
Gait-neutralization. **Left**: Speed signal comparison of an accelerating-cruising-walking sequence before (blue) and after (red) 2 Hz low-pass filtering (criterion measure, gait-neutralized). **Right**: Spectral analysis of a center of mass (COM) speed signal recorded with VICON. Blue line: unfiltered speed signal; red line: 2 Hz low-pass filtered speed signal.

Raw VICON data needs further adjustments in order to serve as an appropriate reference. When humans walk and run, between the heel-strike and mid-stance, the forward speed of the COM decreases and between the mid-stance and toe-off, it increases within each instance of ground contact of each leg [29]. This results in a “true” horizontal speed curve that looks like a sine wave oscillating around the mean horizontal speed (see Fig 4 left).

**Fig 5:**
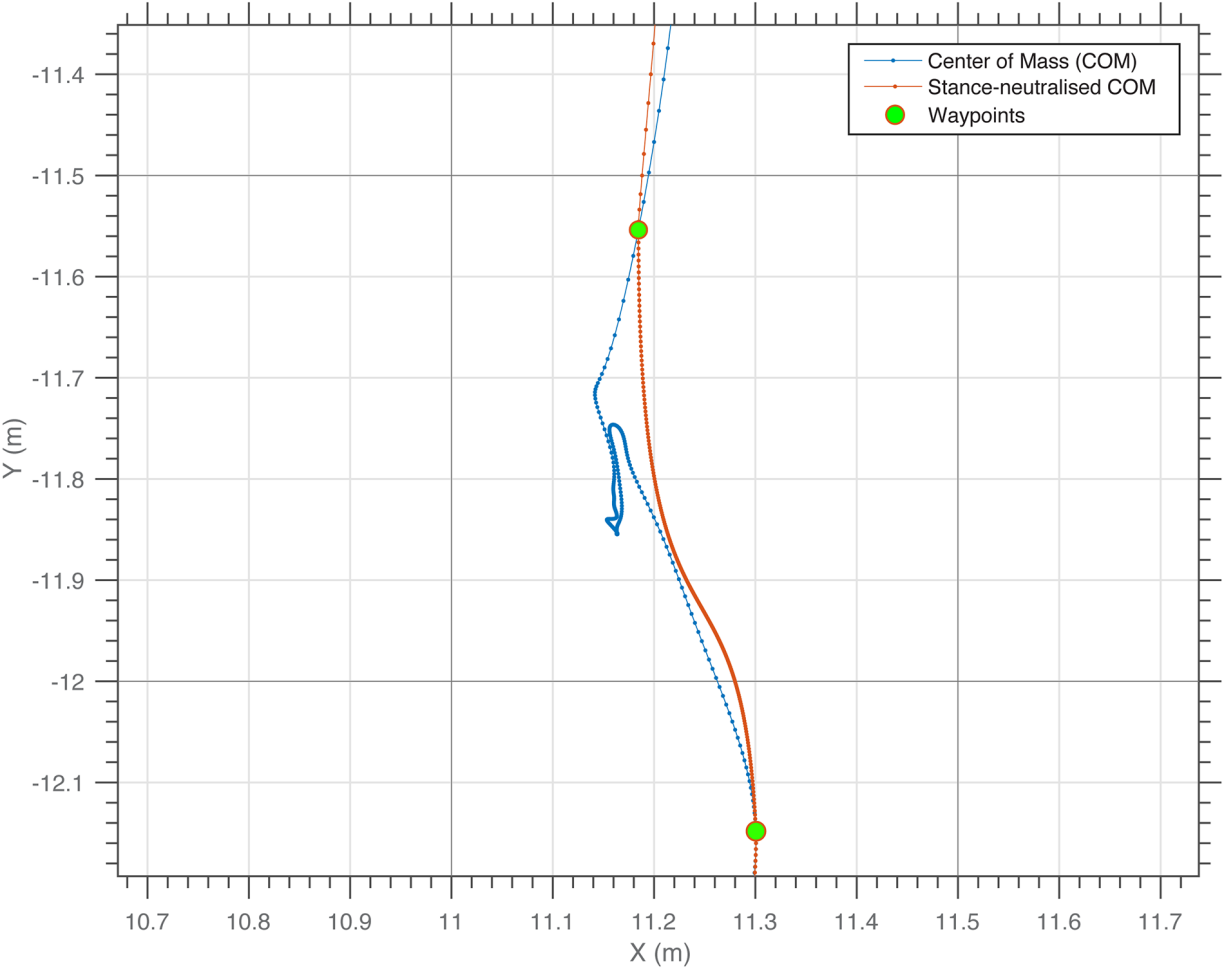
Stance-neutralization with waypoint method. The blue trajectory represents the actual horizontal movement of the center of mass (COM). In the example shown, the athlete moves from south to north and briefly stops in the center. Even if the athlete is standing still (both feet on the ground for a certain time), the COM trajectory would lead to artificial accumulating distances that are measured. The waypoint method (red trajectory) suppresses the micro movements that are not relevant for the gross motion.

Since most EPTS do not have the capability to assess intra-cyclic speed or acceleration fluctuations, a comparison with a gold standard that does have this capability would be “unfair” in the sense that first, there is an increased deviation because intra-cycle speed is not achievable in the case of these systems, and second, EPTS are only meant for assessing the gross movements of players. For this reason, comparisons with “gait-neutralized” sped and acceleration of the gold standard is advisable. To achieve this goal, we studied typical speed signals of football-specific movements through spectral analysis using Fast Fourier Transform (FFT) (Fig 4 right). The occupied bandwidth, as a measurement of the frequency bandwidth that contains 99.0% of the total power of the speed signal, is located at approximately 2 Hz. A further noticeable peak in the spectrum, at approximately 2.5 Hz, most probably corresponds to the intra-cyclic variations of movement speed. Therefore, the gait-neutralized reference speed was calculated using a 4th order 2 Hz Butterworth low pass filter on the raw VICON speed (change in position divided by change in time). The gait-neutralized acceleration was calculated using finite differentiation of the gait-neutralized speed (change in speed divided by change in time). Further analysis of the VICON data showed that the projection of the COM travels considerably even if there is no perceivable movement of a player. This is due natural body sway (see S1 Video for a graphical illustration), or postural changes, which do not result in discernible changes of position. Therefore, the gold standard’s positional data was additionally processed using a “waypoint method” to account for these microscopic movements that are partially detectable only in the case of highly sensitive devices, but not for EPTS, and should be excluded from the assessment of the athlete’s gross motion. The waypoint method assumes that only after a distance traveled between any two tracking points exceeds a certain threshold, typically one step length, these tracking points can be considered for distance calculation. With these remaining points (support points) a new trajectory was calculated using cubic spline interpolation (see Fig 5). We used a threshold of 60.0 cm as our investigations showed that this is a good estimate for the COM displacement during a walk cycle, thus aiming to exclude COM displacements that are smaller than a single step in a way that they are excluded from the measurements of the gross motions of players. It should be stressed here, that the waypoint method was only used to obtain the aggregated distance references, whereas the spatial accuracy (XY-position in space) of each system was validated against the raw VICON positions (4th order 10 Hz Butterworth low pass filter applied to the raw positions).

### Statistical analysis

Accuracy of fundamental XY-position data was estimated by means of the root mean square error (RMSE). Since we also analyzed the error pertaining to speed and acceleration measurements, we distinguish between three types of RMSE: *dRMSE* (m): distance root mean square error (horizontal 2D accuracy); *vRMSE* (m⋅s^-1^): instant speed root mean square error, and *aRMSE* (m⋅s^-2^): instant acceleration root mean square error.

To analyze the accuracy of fundamental (XY-position) and derived (instant speed and acceleration) measures, single-sample t-tests were conducted to determine if the mean of the resulting RMSEs of an individual EPTS was statistically significantly different from zero. Two-tailed paired t-tests were used to compare the aggregated (numerical) metrics derived by the respective EPTS with that derived from the reference system. Inter-system differences in accuracy levels were tested using repeated-measures one-way analysis of variance (ANOVA). Bonferroni’s post hoc analyses were used when significant differences were found. A Shapiro-Wilk test was applied for testing the normality of the residuals and a Levene’s test was used to test the homoscedasticity. In cases where data failed the normality test, non-parametric test procedures were used to analyze the data (Wilcoxon Signed-Ranks test and Kruskal—Wallis test by ranks). Effect sizes (ES) were quantified to indicate the meaningfulness of the differences in the mean values. Cohen’s *d* effect sizes for the t-tests was classified as trivial (0-0.19), small (0.20–0.49), medium (0.50–0.79) and large (>0.80) [30]. Eta squared (*η*^2^) ES for the analysis of variance were classified as small (0.02–0.12), medium (0.13–0.25) and large (>0.26) [30]. Since pre-screening of results revealed skewed error distributions and frequent outliers, descriptive statistics have been presented as the median (Med) and standard deviation (SD). Statistical significance for all calculations was set at *p* <0.05.

### Sample size

Table 1 summarizes the number of single observations included for analysis. The number of observations for each system varied due to organizational reasons, which were mainly caused owing to incomplete data sets, time restrictions and the fact that only one wearable technology could be analyzed at the same time (whereas the VID system recorded all trials—irrespective of which wearable system was measured at the same time). The total number of exercises comprised 26 SSCs, 4 SHUs, and 14 SSGs. The total number of trials included for analysis results from the sum of participating players per exercise (see Table 1). For GPS, LPS, and VID, four, three, and 13 data files contained data gaps. Accordingly, the relative loss of data sets due to measurement errors was 6.3%, 4.2%, and 4.6%, respectively.

**Table 1:** Sample Size.

	GPS	LPS	VID(total)
Sport-specific course (SSC)	6	12	26
Shuttle run (SHU)	20	10	37
Small-sided game (SSG)	38	50	134
**Sum**	**64**	**72**	**197**

Sample size: valid trials (single observations) included for analysis. GPS = Global Positioning System; LPS = Local Positioning System, VID = video system (synonymous with the total number of trials).

## Results

### Fundamental data (position accuracy)

**Fig 6:**
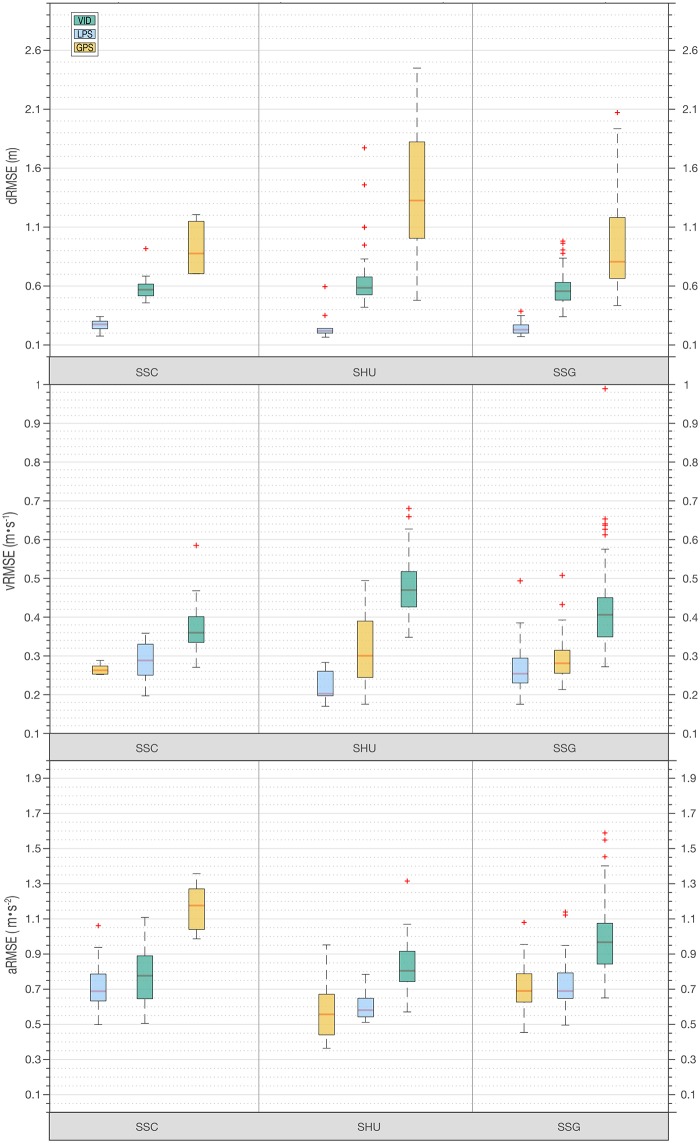
RMSEs by exercise. dRMSE (top), vRMSE (middle) and aRMSE (bottom). Box plots are used based on the five-number summary: minimum, first quartile, median, third quartile, and maximum. The central rectangle spans the first quartile to the third quartile. A red line inside the rectangle represents the median and the whiskers above and below the box show the locations of the maximum and minimum value. Red crosses indicate outliers. GPS = Global Positioning System; LPS = Local Positioning System, VID = video system; dRMSE = distance root mean square error; vRMSE = velocity root mean square error; aRMSE = acceleration root mean square error.

Table 2 and Fig 6 report the measurement error of EPTS in the respective category. Overall, smallest errors of fundamental spatial accuracy (dRMSE) were achieved by the radar-based LPS system (SSC: 27±5 cm; SHU: 22±13 cm; SSG: 23±5 cm; pooled: 23±7 cm), followed by the image-based VID system (SSC: 57±9 cm; SHU: 59±28 cm; SSG: 56±12 cm; pooled: 56±16 cm), and the GPS system (SSC: 88±22 cm; SHU: 133±54 cm; SSG: 81±51 cm; pooled: 96±49 cm). GPS showed noticeable exercise-dependent fluctuations in spatial accuracy. In particular, GPS demonstrated lower spatial accuracy during the shuttle runs (133±54 cm). For VID, we found significant differences in the X and Y dRMSE accuracy (X: 28±13 cm, Y: 50±15 cm) [F(1, 392) = 247.40, p<.001]. Post hoc analysis of the ANOVA revealed no homogeneous subsets, implying that the spatial error (dRMSE) differs significantly between all tested systems.

**Table 2:** RMSE results.

		LPS		GPS		VID		ANOVA		
		Median	±*SD*	Median	±*SD*	Median	±*SD*	p	ES	Sign. diff. groups
dRMSE (m)	Sport-specific course (SSC)	0.27	*0.05*	0.88	*0.22*	0.57	*0.09*	***	large	ALL
	Shuttle run (SHU)	0.22	*0.13*	1.33	*0.54*	0.59	*0.28*	***	large	ALL
	Small-sided game (SSG)	0.23	*0.05*	0.81	*0.41*	0.56	*0.12*	***	large	ALL
vRMSE (m⋅s^-1^)	Sport-specific course (SSC)	0.35	*0.06*	0.32	*0.01*	0.41	*0.07*	**	large	GPS&VID / LPS&VID
	Shuttle run (SHU)	0.31	*0.04*	0.39	*0.08*	0.52	*0.08*	***	large	GPS&VID / LPS&VID
	Small-sided game (SSG)	0.36	*0.06*	0.39	*0.06*	0.47	*0.08*	***	large	GPS&VID / LPS&VID
	Standing (pooled)	0.34	*0.17*	0.18	*0.20*	0.23	*0.26*	***	medium	GPS&LPS / VID&LPS
	Low speed (pooled)	0.26	*0.07*	0.26	*0.13*	0.33	*0.12*	***	medium	GPS&VID / LPS&VID
	Moderate speed (pooled)	0.25	*0.07*	0.27	*0.07*	0.43	*0.10*	***	large	GPS&VID / LPS&VID
	Elevated speed (pooled)	0.34	*0.12*	0.37	*0.19*	0.49	*0.24*	***	small	GPS&VID / LPS&VID
	High speed (pooled)	0.39	*0.13*	0.37	*0.25*	0.50	*0.30*			
	Very high speed (pooled)	0.37	*0.12*	0.39	*0.13*	0.61	*0.43*	*	medium	GPS&VID / LPS&VID
aRMSE (m⋅s^-2^)	Sport-specific course (SSC)	0.69	*0.16*	1.18	*0.14*	0.78	*0.16*	***	large	GPS&LPS / GPS&VID
	Shuttle run (SHU)	0.58	*0.10*	0.56	*0.17*	0.80	*0.15*	***	large	GPS&VID / LPS&VID
	Small-sided game (SSG)	0.69	*0.13*	0.69	*0.14*	0.97	*0.19*	***	large	GPS&VID / LPS&VID

Presented as median ± standard deviation (SD). Inter-system differences in accuracy levels were tested using repeated-measures one-way analysis of variance (ANOVA). *p* values are presented as * (*p* ≤ 0.05), ** (*p* ≤ 0.01) and *** (*p* ≤ 0.001). *η*^2^ effect sizes (ES) for the analysis of variance were classified as *small* (0.02–0.12), *medium* (0.13–0.25) and *large* (>0.26). Homogeneous subsets are listed if Bonferroni’s post hoc analysis did not result in significant difference between individual groups. GPS = Global Positioning System; LPS = Local Positioning System, VID = video system; dRMSE = distance root mean square error; vRMSE = velocity root mean square error; aRMSE = acceleration root mean square error.

### Derived data (instant speed and acceleration)

Lowest errors in vRMSE were achieved by the radar-based LPS system (SSC: 0.29±0.05 m⋅s^-1^; SHU: 0.20±0.04 m⋅s^-1^; SSG: 0.25±0.06 m⋅s^-1^; pooled: 0.25±0.06 m⋅s^-1^), followed by GPS (SSC: 0.26±0.01 m⋅s^-1^; SHU: 0.30±0.09 m⋅s^-1^; SSG: 0.28±0.06 m⋅s^-1^; pooled: 0.28±0.07 m⋅s^-1^) and VID (SSC: 0.36±0.07 m⋅s^-1^; SHU: 0.47±0.08 m⋅s^-1^; SSG: 0.41±0.08 m⋅s^-1^; pooled: 0.41±0.08 m⋅s^-1^) (Table 2). It is also apparent that each system’s speed accuracy depends on the respective exercise. Whereas LPS presented the lowest speed error during the shuttle run trials, GPS and VID showed the lowest speed error during the sport-specific course trials. The point that all systems have in common is that the speed error increases as the speed increases.

Overall, it is apparent that the acceleration error (aRMSE) follows a similar pattern as the vRMSE categories. Due to the necessary step of derivation, an increase in the overall error is recognizable for all systems. For GPS, the error of the instantaneous acceleration (aRMSE) is twice as high during the sport-specific course (1.18±0.14 m⋅s^-2^) when compared to the shuttle run (0.56±0.17 m⋅s^-2^).

Post hoc analysis of the ANOVA revealed notably frequent homogeneous subsets of GPS and LPS, implying that the vRMSE and aRMSE of GPS and LPS did not differ significantly (with the only exception of aRMSE for SSCs and vRMSE for standing (see Table 2)).

### Sport-specific course

Results of the specific categorization into fundamental movement patterns during the SSC trials are presented in Table 3. The point that all the systems have in common is that dRMSE, vRMSE, and aRMSE were lowest during low speed location changes. Compared to GPS and LPS, VID showed significantly lower speed accuracy values during linear sprint exercises (15 m sprint into 5 m acceleration and backward into forward sprints), which were both aligned at a 90° angle (perpendicular to the camera view). However, in the opposite direction, (505 agility test, movements parallel to the camera view), VID showed smaller errors (0.32±0.23 m⋅s^-1^) than both LPS (0.51±0.07 m⋅s^-1^) and GPS (0.53±0.11 m⋅s^-1^).

**Table 3:** Results of the sport-specific course SSC.

		LPS		GPS		VID		ANOVA		
		Median	±*SD*	Median	±*SD*	Median	±*SD*	p	ES	Sign. diff. groups
dRMSE (m)	Low speed location change	0.21	*0.04*	0.79	*0.18*	0.45	*0.09*	***	large	LPS&GPS / LPS&VID
	15m sprint / 5m deceleration	0.34	*0.16*	0.99	*0.29*	0.63	*0.71*	**	large	LPS&GPS / LPS&VID
	Backward / forward sprint	0.29	*0.05*	1.14	*0.42*	0.70	*0.22*	***	large	LPS&GPS / LPS&VID
	505 agility test	0.42	*0.12*	0.99	*0.15*	0.92	*0.20*	***	large	LPS&GPS / LPS&VID
	90¡ turns	0.45	*0.10*	0.92	*0.16*	0.75	*0.07*	***	large	LPS&GPS / LPS&VID
	Curved run I (towards)	0.41	*0.17*	1.21	*0.32*	0.70	*0.15*	***	large	LPS&GPS / LPS&VID
	Curved run II (away)	0.48	*0.12*	1.27	*0.50*	0.60	*0.18*	***	large	LPS&GPS / GPS&VID
vRMSE (m⋅s^-1^)	Low speed location change	0.20	*0.05*	0.16	*0.02*	0.22	*0.05*	**	medium	LPS&GPS / GPS&VID
	15m sprint / 5m deceleration	0.32	*0.23*	0.27	*0.04*	0.63	*0.39*	***	large	LPS&VID / GPS&VID
	Backward / forward sprint	0.38	*0.07*	0.25	*0.04*	0.63	*0.20*	***	large	LPS&VID / GPS&VID
	505 agility test	0.51	*0.07*	0.53	*0.11*	0.32	*0.23*			NONE
	90¡ turns	0.44	*0.10*	0.35	*0.09*	0.79	*0.15*	***	large	LPS&VID / GPS&VID
	Curved run I (towards)	0.47	*0.14*	0.52	*0.10*	0.52	*0.09*			NONE
	Curved run II (away)	0.46	*0.14*	0.54	*0.19*	0.46	*0.15*			NONE
aRMSE (m⋅s^-2^)	Low speed location change	0.49	*0.15*	0.93	*0.20*	0.44	*0.14*	***	large	LPS&GPS / GPS&VID
	15m sprint / 5m deceleration	0.93	*0.86*	1.22	*0.35*	1.33	*0.70*			NONE
	Backward / forward sprint	0.87	*0.14*	1.20	*0.18*	1.23	*0.42*	**	large	LPS&GPS / LPS&VID
	505 agility test	1.24	*0.22*	2.07	*0.37*	0.85	*0.50*	***	large	LPS&GPS / GPS&VID
	90¡ turns	1.34	*0.37*	1.45	*0.33*	1.65	*0.49*			NONE
	Curved run I (towards)	0.94	*0.16*	1.55	*0.24*	1.02	*0.28*	**	large	LPS&GPS / GPS&VID
	Curved run II (away)	1.04	*0.43*	1.61	*0.35*	0.78	*0.26*	**	large	LPS&GPS / GPS&VID

Presented as median ± standard deviation (SD). *p* values are presented as * (*p* ≤ 0.05), ** (*p* ≤ 0.01) and *** (*p* ≤ 0.001). *η*^2^ effect sizes (ES) for the analysis of variance were classified as *small* (0.02–0.12), *medium* (0.13–0.25) and *large* (>0.26). Homogeneous subsets are listed if Bonferroni’s post hoc analysis did not result in a significant difference between individual groups. GPS = Global Positioning System; LPS = Local Positioning System, VID = video system; dRMSE = distance root mean square error; vRMSE = velocity root mean square error; aRMSE = acceleration root mean square error.

#### Results of key performance indicators (KPI)

The percentage difference in KPIs between the respective EPTS and the criterion measure are presented in Table 4.

**Table 4:** Results of key performance indicators (KPI).

		LPS					GPS					VID				
Test	Metric	MeanGS	RMSE	RMSE%	p	ES	MeanGS	RMSE	RMSE%	p	ES	MeanGS	RMSE	RMSE%	p	ES
SSC	Standing (m)	*0.81*	0.68	83.94	**	*medium*	*0.86*	3.68	429.85	*	*large*	*2.52*	5.89	234.23	***	*large*
	Low speed (m)	*102.93*	5.40	5.24	-	-	*82.30*	6.30	7.66	*	*trivial*	*96.96*	11.24	11.60	***	*small*
	Moderate speed (m)	*87.80*	8.04	9.15	-	-	*92.55*	7.94	8.58	-	-	*92.22*	15.52	16.83	**	*small*
	Elevated speed (m)	*51.80*	7.22	13.94	**	*large*	*43.80*	6.39	14.58	-	-	*45.86*	19.16	41.78	*	*small*
	High speed (m)	*52.76*	11.60	21.98	***	*medium*	*51.61*	9.35	18.11	-	-	*51.36*	16.34	31.82	-	-
	Very high speed (m)	*18.55*	5.31	28.65	-	-	*18.51*	9.46	51.12	*	*large*	*13.18*	12.91	97.94	-	-
	High acceleration (m)	*17.56*	6.60	37.58	***	*large*	*14.91*	9.71	65.14	*	*large*	*24.35*	8.50	34.90	-	-
	High deceleration (m)	*24.83*	3.93	15.82	-	-	*23.55*	10.94	46.46	-	-	*13.63*	4.35	31.94	-	-
	Total distance (m)	*314.64*	7.31	2.32	***	*small*	*289.63*	3.54	1.22	-	-	*301.59*	3.59	1.19	*	-
	Top speed (m⋅s^-1^)	*7.61*	0.34	4.51	-	-	*7.69*	0.31	4.03	*	*large*	*7.50*	0.51	6.81	-	-
SHU	Standing (m)	*0.15*	0.18	116.23	*	*large*	*0.09*	0.73	856.39	***	*large*	*0.38*	2.28	597.83	***	*large*
	Low speed (m)	*25.86*	1.30	5.01	-	-	*21.69*	12.41	57.22	***	*large*	*31.52*	13.69	43.42	***	*large*
	Moderate speed (m)	*440.09*	4.98	1.13	-	-	*427.69*	26.93	6.30	-	-	*389.35*	51.50	13.23	***	*small*
	Elevated speed (m)	*1.63*	3.38	207.14	-	-	*51.35*	39.81	77.53	*	*small*	*28.43*	73.71	259.25	***	*small*
	High speed (m)	-	-	-	-	-	-	-	-	-	-	-	-	-	-	-
	Very high speed (m)	-	-	-	-	-	-	-	-	-	-	-	-	-	-	-
	High acceleration (m)	*5.12*	3.68	71.85	**	*large*	*9.26*	3.24	34.99	-	-	*14.06*	10.87	77.30	**	*small*
	High deceleration (m)	*4.67*	4.84	103.66	**	*large*	*12.09*	7.33	60.60	**	*small*	*3.61*	2.39	66.08	*	*trivial*
	Total distance (m)	*466.88*	3.45	0.74	-	-	*485.94*	21.44	4.41	-	-	*446.17*	19.77	4.43	**	*trivial*
	Top speed (m⋅s^-1^)	*4.07*	0.46	11.32	-	-	*4.44*	0.22	5.01	-	-	*4.34*	0.71	16.37	***	*medium*
SSG	Standing (m)	*0.29*	0.27	95.00	***	*large*	*0.27*	3.27	1194.41	***	*large*	*0.40*	1.83	455.30	***	*large*
	Low speed (m)	*34.11*	2.73	7.99	-	-	*42.46*	7.80	18.37	***	*small*	*36.92*	6.21	16.83	**	*trivial*
	Moderate speed (m)	*106.87*	6.59	6.16	***	*trivial*	*156.89*	5.87	3.74		-	*113.20*	10.34	9.13	-	-
	Elevated speed (m)	*13.74*	2.98	21.67	-	-	*22.20*	8.58	38.65	***	*small*	*16.33*	8.05	49.30	***	*trivial*
	High speed (m)	*5.91*	2.59	43.77	-	-	*4.12*	4.01	97.44	***	*medium*	*5.72*	5.58	97.62	-	-
	Very high speed (m)	-	-	-	-	-	-	-	-	-	-	-	-	-	-	-
	High acceleration (m)	*3.68*	3.05	82.87	***	*medium*	*5.64*	2.83	50.25	***	*small*	*6.41*	5.83	90.87	***	*medium*
	High deceleration (m)	*3.26*	2.34	71.92	***	*medium*	*9.12*	8.50	93.25	***	*medium*	*2.96*	3.00	101.11	***	*medium*
	Total distance (m)	*153.38*	6.05	3.95	***	*trivial*	*224.10*	4.90	2.18	**	*trivial*	*165.00*	4.60	2.79	***	*trivial*
	Top speed (m⋅s^-1^)	*4.81*	0.34	7.09	*	*trivial*	*5.47*	0.33	6.08	***	*small*	*4.86*	0.42	8.64	-	-

Deviation from the criterion standard presented as root mean square error (RMSE) and the percentage RMSE (RMSE in relation to the mean total distance in the respective category measured by the gold standard *MeanGS*). The table shows accuracy results for the covered distances in various intensity zones as well as the measured peak speed. *p* values are presented as * (*p* ≤ 0.05), ** (*p* ≤ 0.01) and *** (*p* ≤ 0.001). Cohen’s *d* effect sizes (ES) for the t-tests was classified as trivial (0-0.19), small (0.20–0.49), medium (0.50–0.79) and large (>0.80). GPS = Global Positioning System; LPS = Local Positioning System, VID = video system, SSC = sport-specific course; SHU = shuttle run; SSG = small-sided game.

## Discussion

Results showed that largest accuracy differences between EPTS were present in the first data category (fundamental XY-position in space). In particular, LPS had higher accuracy than both VID and GPS for measuring an athlete’s position in space. However, our results also revealed that in the second category (instant speed and acceleration) errors of GPS are comparable to those of LPS, most likely related to the fact that GPS uses two fundamentally different measurement principles to determine an athlete’s position and speed. In the third data category (KPIs), differences between technologies were not as pronounced as in the first and second data category, yet all technologies had in common that the magnitude of the error increased as the speed of the tracking object increased.

### Position accuracy

The radar-based LPS system demonstrated the highest spatial accuracy with a dRMSE ranging from 22 cm (SHU) to 27 cm (SSC) (see Table 2). These findings are in accordance with previous research by Ogris et al. [8] (23 cm) and Siegle et al. [17] (24 cm). The sport-specific course has, however, also revealed that the spatial accuracy of the LPS system is dependent on instantaneous dynamics. In particular, fast changes of direction can lead to a significant increase of the spatial error (e.g. 0.45 cm during 90° turns, see Table 3). Rapid speed and direction changes seem to be a challenge for the underlying Kalman filter, which is generally based on linear dynamical systems, thus suppressing rapid movement changes.

The only previous study that analyzed the spatial accuracy of the VID system reported an error of 73 cm dRMSE [17] (vs. 56-59 cm in the present study, see Table 2). Such a difference could be caused by either technological advancements in camera gear, different viewing angles, or the used criterion reference (LAVEG vs. VICON). For VID, we found significantly higher spatial errors in the 505 agility test of the sport-specific course (Table 3). Since players tend to lower their upper body to counteract accelerations occurring at the turning point, we assume that the visual tracking algorithm detects the center of the athletes’ body at a lower height, thus leading to a spatial position shift in the vertical Y-axis.

To the best of our knowledge, information about the spatial accuracy of sport-specific GPS systems has not been reported prior to this study. This could be due to the fact that GPS systems are predominantly used to evaluate physical performance metrics (rather than spatial/tactical behavior). Nevertheless, it is incomprehensible why only limited information is available on the spatial accuracy of GPS systems, especially against the background that various studies made use of GPS coordinates to analyse spatial motion behaviour (e.g. position-specific centroids, team centroids and distance between centroids [24, 25]), as well as the fact that several commercial GPS systems determine distance metrics via differentiation of position data [21]. This study shows that the average spatial measurement error of GPS was 96 cm, almost twice as high as that of its nearest competitor VID (56 cm).

### Instantaneous speed and acceleration accuracy

Considering that GPS exhibited the highest spatial errors, one would think that this error pattern should exert its influence on the vRMSE/aRMSE categories. Contrastingly, it is found that GPS speed errors were not significantly different from those of LPS (see Table 2). Whereas the vision-based and radar-based technology utilize differentiation of position data over time for speed determination, most commercially available GPS systems circumvent the problem of error propagation from fundamental to derived data by using two completely different measurement principles. Modern GPS systems can determine speed by measuring the rate of change in the satellites’ electromagnetic signal frequency, also known as the Doppler effect [31, 32]. Doppler measurements are immune to cycle slips (temporary signal anomalies or low signal-to-noise ratio caused by obstructions such as buildings, trees, etc.) [33]. Thus, research works dealing with GPS speed measurement reveal that using GPS Doppler measurements can provide greater speed accuracy than indirect measurement, which is based on error-prone position data [34]. As a consequence, despite comparably inferior spatial accuracy values, GPS systems are capable of measuring instantaneous speed, and consequently acceleration, with comparatively higher accuracy.

As depicted in the results, errors of the VID system were lower in movements in the X-axis when compared to movements in the Y-axis. Thus, lowest vRMSE errors for section 3 (505) were achieved by VID. This specific test was carried out in parallel alignment to the camera view (X-axis). Apart from that, it is apparent that instant speed an acceleration errors of the LPS and GPS technology are fairly consistent whereas the errors of the video technology have proven to be considerably higher. These results demonstrate the importance of the most accurate possible detection of position in space. Any inaccuracy on the fundamental data (XY-positions) will otherwise lead to increased error propagation in the derived data category (instantaneous speed and acceleration).

### KPI accuracy

Overall, lowest deviations can be observed in the total distance category. RMSE% ranged from 1.2% (VID during SSC) to 4.9% (GPS during SSGs). These differences are in line with previous literature on GPS (1.9%) [35] and LPS (1.6—2.0%) [7, 22]. Given a total distance of approximately 11.4 ± 1.0 km in professional soccer matches [36], an error of 4.9% would correspond to a discrepancy of 560 m, which in turn is more than half a standard deviation (1.0 km). It is therefore questionable to what extent EPTS with an apparently small error of e.g. 4.9% for total distance can sufficiently describe the performance hierarchy between players. In agreement with previous studies [6, 15], we found evidence that GPS units are capable of accurately measuring distances with low and moderate speed (see Table 4), whereas they still have problems with regard to tracking movements involving high-speed direction changes (e.g. 90-180° turns, see Table 3). GPS had the lowest sampling rate in this study (GPSports 15 Hz units are actually 5 Hz with interpolated data). Our results again confirm that a 5 Hz sampling rate only partially captures high-intensity movements involving frequent changes of direction. Similar findings have been identified by previous GPS validation studies [10, 15]. The generally high deviations in the lowest speed category of all systems (standing, <1 km⋅h^-1^) can be attributed to the fact that standing phases practically never occurred during the exercises, and thus the values for the total standing distance were considerably low. Minor differences could therefore lead to high deviations. The same applies to high-intensity categories such as high speed distance or high acceleration distance. It should also be noted that the RMSE% increases significantly as the movement intensity increases. This characteristic error pattern is particularly obvious in the case of high-speed categories. Considering that the percentage deviation increases considerably in these relevant performance categories (e.g. high-speed distance during small-sided games: RMSE% ranged from 43.8% (LPS) to 97.6% (VID)), the present results confirm that to this day EPTS may not be accurate enough to measure high-speed and acceleration distances with a reasonable degree of accuracy [13]. The RMSE in the peak speed category ranged between 0.22 m⋅s^-1^ (GPS during shuttle runs) and 0.71 m⋅s^-1^ (VID during shuttle runs). These values reveal the technology-dependent accuracy variations of the VID system. As the movement direction of the shuttle run was conducted in the vertical (perpendicular) camera axis (Y-axis), VID tended to overestimate the peak speed during shuttle runs.

### Limitations

It is regrettable that at the time being, there is no gold standard for a full-size pitch of team-based sports. Since the natural field of application of EPTS are official matches, this leads to the fact that they might not be validated in the scenarios that are most relevant to them.

We could provide optimum environmental conditions for LPS and near-optimum conditions for GPS but could only meet the minimum requirements in case of the the VID system. It can be assumed that results of the VID system improve under optimum conditions such as in stadiums with steep stands in close proximity to the pitch.

It is worth noting that our results are based on untreated raw data, as provided by the manufacturer’s proprietary software. Therefore, it is to be expected that the validity of the tested EPTS could be further improved by additional data filtering procedures.

Finally, this study did not examine the inter-unit agreement, i.e. systematic or random differences between different sensors in GPS and LPS systems. This is important though, in case valid comparisons between different players and sessions are of interest in the sensor-based systems [15].

## Conclusion

Collectively, results of this study revealed that largest differences between EPTS occurred at the spatial accuracy, whereas speed and acceleration errors of GPS were comparable to those of LPS. Yet one important insight in this regard is the noticeably large error margin in the third data category (accuracy of KPIs) that is independent of the respective system or technology, which we are still facing in EPTS in general. Especially in KPI categories that might have a high impact on practical decisions, such as high speed performance indicators, we found significant deviations from the gold standard. Thus, the primary aim of future activities should be aimed at diminishing these inherent errors. Until then, it is recommended that practitioners do not make direct comparisons between KPIs collected by different EPTS. Since there are typically different systems at work in competition and training, we encourage any development toward a standardization of internal algorithms. In case there is no hint available at different operational definitions for filtering techniques or KPIs in different systems, this means the sports practice is led astray. For the time being, a consequence in this regard is to conduct comparisons between EPTS on the level of XY-data, instantaneous speed, and acceleration data, in addition to merely comparing calculated KPIs.

## Supporting information

S1 Video Body sway visualization. Exemplary 3D animation of the center of mass (COM) displacement. Despite being static (the whole body is not traveling any distance in the conventional sense), the animation demonstrates that COM is constantly in motion, thus leading to unintended accumulation of travel distance. This example demonstrates the need for a distance calculation method that compensates this effect.
(MP4)

S2 Video Visualization of the test procedures. Exemplary animation of the scouting video and the motion capture data. Red dots and lines indicate the center of mass (COM) that was projected to the ground plane. Colored lines represent the position as reported by the respective tracking technology.
(MP4)

S1 Dataset Calibration run. (CSV)

S2 Dataset System database. (XLSX)
